# Manganese Ferrite/Guava Leaf Nano-Bio Composite for Adsorptive Removal of Methylene Blue Dye from Water

**DOI:** 10.3390/molecules31101754

**Published:** 2026-05-20

**Authors:** Noufal Komby Abdulla, Elham A. Alzahrani, Ghaida H. Munshi, Abeer Mohammed AL-Balawi, Salwa D. Al-Malwi, Naha Meslet Alsebaii, Sumbul Hafeez, Seungdae Oh, Saif Ali Chaudhry

**Affiliations:** 1 Environmental Chemistry Research Laboratory, Department of Chemistry, Jamia Millia Islamia, New Delhi 110025, India; noufal78666@gmail.com; 2 Department of Chemistry, College of Science, University of Ha’il, Ha’il 81451, Saudi Arabia; elhama.alzahrani@gmail.com; 3 Department of Chemistry, College of Science, University of Jeddah, Jeddah 21959, Saudi Arabia; gmunshi@uj.edu.sa; 4 Department of Chemistry, University Duba College, University of Tabuk, Tabuk 71491, Saudi Arabia; ab_albalawi@ut.edu.sa; 5 Department of Chemistry, College of Science, Northern Border University, Arar 91431, Saudi Arabia; salwa.almaalawi@nbu.edu.sa; 6 Department of Chemistry, Faculty of Science, King Abdulaziz University, P.O. Box 80200, Jeddah 21589, Saudi Arabia; nalsebaii@kau.edu.sa; 7 Department of Civil and Environmental Engineering, Villanova University, Villanova, PA 19085, USA; 8 Department of Civil Engineering, College of Engineering, Kyung Hee University, Yongin 17104, Republic of Korea

**Keywords:** environmental remediation, water, hybrid biosorbent, adsorption kinetics, isotherm modeling

## Abstract

In this study, manganese ferrite was grown on the surface of a low-cost powder substrate of a guava leaf using the co-precipitation method. The resulting material was characterized using various spectroscopic and microscopic techniques. The composite was formed through the electrostatic and non-electrostatic interactions between the manganese ferrite nanoparticles, and the functional groups present on the guava leaf substrate; consequently, a high content of functional groups was observed in the synthesized composite through the Fourier transform infrared spectroscopy. The average size of the nanoparticles grown on the guava leaf substrate was determined to be between 3 and 5 nanometers. The synthesized composite material was utilized for adsorption applications, employing Methylene blue dye as a model adsorbate. Methylene blue was removed from the aqueous solutions under various conditions—including variations in the pH, contact time, temperature, and concentration. Under optimal conditions, it was observed that an adsorbent dosage of 2 g L^−1^ was capable of removing approximately 99% of the dye from a 10 mg L^−1^ dye solution at pH 7. The dye removal efficiency (%) decreased with the increasing temperature, indicating an exothermic process; this was further confirmed by the thermodynamic parameter analysis (specifically, the change in enthalpy, or ΔH), which yielded a negative value. Gibbs Free Energy (ΔG) also yielded a negative value, signifying the feasibility and spontaneity of the adsorption process. In this study, the adsorption process followed the Freundlich isotherm model, with the value of ‘*n*’ falling between 1 and 10, which is indicative of heterogeneous adsorption. The adsorption kinetics were determined to follow a pseudo-second-order model, and the overall rate-limiting step of the process was identified as intraparticle diffusion. To assess the sustainability and stability of the adsorbent, regeneration and reusability experiments were conducted. The results revealed that the modified guava leaf performed effectively for up to five cycles, achieving an adsorption efficiency of approximately 24% after the final cycle. Thus, the developed adsorbent proved to be an effective material for the removal of Methylene blue dye.

## 1. Introduction

Everyone is well aware that human life depends on water; however, due to a growing population and industrialization, this water is becoming increasingly contaminated [1]. A primary cause of this pollution is the use of dyes [2]. Many industries, such as paint [3], paper [4], plastics [5], and textile [6], utilize coloring agents (dyes). These dyes are toxic and pose serious health risks not only to the human body, but also to aquatic flora and fauna [7]. Among these, ‘Methylene blue’ (MB) is a dye primarily employed in various industrial applications [8]. MB contains an ‘azo group’ and possesses a highly complex molecular structure [9]; it obstructs the penetration of sunlight into the water [10], thereby adversely affecting the water’s ecological quality. Furthermore, exposure to MB through contaminated water has serious effects on human health [11]. Given the rising concentration of this dye in water, it is imperative that it be removed immediately [12]. Various processes categorized into physical [12], chemical [13], and biological methods [14] have been employed to remove such dyes from water. Upon comparing these diverse approaches, it can be concluded that ‘adsorption’ stands out as the most effective method, being a simple, user-friendly, and cost-efficient process [15].

Materials ranging from charcoal [16] to sand [17] and clay [18] have historically been utilized for adsorption purposes—a progression that has evolved to encompass biochar [19] and activated carbon [20]. Over the past decade, nano-sized adsorbents such as nanoparticles (NPs) have been frequently employed [21]; however, virgin NPs typically exhibit low adsorption capacity [22,23]. In recent times, numerous efforts have been undertaken to enhance the efficiency of NPs, including nanohybrid composite [24,25].

The use of nanohybrid composites in water purification has been identified as a promising candidate due to their significant reusability and improved processing, as well as targeted pollutant removal capabilities over virgin nanomaterials [24,25,26]. The fabrication of the organic-inorganic nanohybrid composites for wastewater treatment applications has been the subject of extensive research [25,26]. Nanohybrid composites further have received immense attention because of their exceptional mechanical and chemical stability as well as their superior physicochemical characteristics [25].

Notably, ferrite [27,28] and iron oxide [29,30] nanohybrid composites have been extensively developed and effectively studied for dye removal from aqueous solutions. These composites are being investigated as promising candidates due to their facile synthesis, cost-effectiveness, and environmental compatibility [27,28,29,30,31]. Among these, nanohybrid composites based on activated carbon and biochar have been shown to be promising candidates as efficient adsorbents [30,31]; however, the development of biochar as well as activated carbon requires complex procedures involving high-temperature processing and energy-intensive equipment such as a furnace [32].

To overcome these challenges, research is significantly focused on identifying substantial alternatives to biochar and activated carbon for the development of efficient nanohybrid composites.

Recent studies have explored plant-based materials such as leaves [33,34], seeds [35,36], gum [37], and mucilage [38] for the development of nanohybrid composites without converting them into biochar or activated carbon. This approach has introduced a novel perspective in material research by leveraging the inherent properties of plant-based materials. It has been observed that plants possess a cellulosic surface with abundant functional entities that are capable of interacting with the metal ions during the composite’s synthesis process [39]. Furthermore, the synthetic material’s structural integrity is improved by the stabilizing and capping effect of phytochemicals found in the plants [39]. The resultant nanohybrid composites are categorized as nano-bio-composites due to their distinct properties, such as the high density of functional sites, reduced particle size, and economical production cost [39]. These materials demonstrated versatility in applications including photocatalysis [40], antifungal activity [41], and especially adsorption processes [39].

Notably, the functionalized NPs synthesized by bioprecipitation with only plant extracts and without the plant residue have been extensively explored [41]. However, when compared to the nano-bio-composite synthesized without discarding plant residues, the related expenses of such NPs are comparatively higher [39]. This makes nano-bio-composites a more cost-effective and technologically advanced alternative.

This study also explores similar plant-material-based nanohybrid composites. In the search for suitable plant materials, key considerations include global availability, abundance, low cost, and edibility, which contribute to lower toxicity risks [42]. Guava leaves (GL), which are easily available, fulfill these requirements [42]; therefore, they have been successfully utilized in this study. GL have also been utilized in previous studies, wherein an organic-inorganic γ-Fe_2_O_3_/GL [43] and MnO_2_/GL [44] hybrid nanocomposite was synthesized via a straightforward, cost-effective, and eco-friendly co-precipitation method. The study highlights the potential of GL as an efficient and sustainable platform for developing nano-bio-composites with promising applications in wastewater treatment and beyond.

This work aims to synthesize and employ GL-based manganese ferrite nano-hybrid composite, MnFe_2_O_4_/GL, using the co-precipitation technique in search of a cost-effective and efficient approach. MnFe_2_O_4_ exhibits superior properties, as well as biocompatibility, compared to other metal oxide NPs [45,46,47,48]. Due to the presence of the manganese ions, its adsorption ability is high [45,46]. Furthermore, it facilitates photocatalytic degradation, which can aid in completely degrading and removing residual dye content from water following the initial adsorption process [48,49]. According to the existing literature [50], the surface properties of MnFe_2_O_4_ can be rendered highly tunable. In light of all these features, an upgrade was undertaken to transition from Fe_2_O_3_ to MnFe_2_O_4_.

The physicochemical properties of the synthesized nanohybrid composites were subjected to an in-depth analysis and a systematic study to comprehensively evaluate their potential for the removal of MB dye from aqueous media. According to the current state of research, no previous studies have reported the synthesis of magnetic MnFe_2_O_4_/GL using the co-precipitation method and its application in the adsorption of MB. The adsorption efficiency of MnFe_2_O_4_/GL was systematically investigated by analyzing key parameters such as adsorbent dosage, initial dye concentration, solution pH, and contact time. The collected data were further evaluated to elucidate the adsorption mechanism along with detailed kinetics and isotherm studies to provide a comprehensive understanding of MB removal using MnFe_2_O_4_/GL composites.

## 2. Experimental

### 2.1. Material and Methods

The precursors used to prepare the MnFe_2_O_4_/GL composite, manganese chloride (MnCl_2_) and ferric chloride (FeCl_3_), were purchased from Merck Ltd., Maharashtra, India. Methylene blue (MB) dye was purchased from Sigma-Aldrich, Karnataka, India. Guava leaves were collected from the vicinity of the Department of Chemistry, Gate No. 8, Jamia Millia Islamia, New Delhi, India. Hydrochloric acid (HCl) and sodium hydroxide (NaOH) were purchased from Merck Ltd., Maharashtra, India, and were used for the adjustment of the pH during preparation and adsorption experiments. All reagents were utilized without further purification, and double-distilled (DD) water was used for all experiments.

### 2.2. Preparation of MnFe_2_O_4_/GL

In this study, guava leaves were used as the organic material. The guava leaves were thoroughly washed with tap water, followed by multiple rinses with DD to eliminate any impurities. After numerous washes, leaves were dried at 90 °C for 48 h under a vacuum oven. The dried leaves were ground into a powder, sieved (60 mesh), and referred to as guava leaves (GL) powder. The MnFe_2_O_4_/GL hybrid composite was prepared by using a simple co-precipitation method [51].

**Scheme 1:**
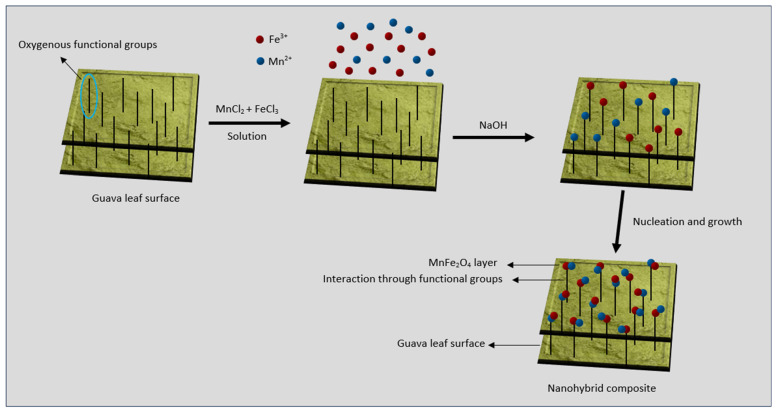
Schematic diagram of preparation of MnFe_2_O_4_/GL.

Briefly, GL powder (1.0 g) was suspended in DD (300 mL) using an ultrasonic bath for ultrasonication. Following dispersion, 50 mL of each precursor salt solution (0.05 M of MnCl_2_ and 0.1 M of FeCl_3_) was added to the dispersed mixture at a constant stirring of 700 rpm for 60 min at 60 °C. The pH of the solution was maintained at 10.5 by gradually adding 4 M NaOH solution under continuous stirring. As the pH approached 10.5, the color of the solution turned yellowish-brown, indicating the onset of precipitation and followed by an additional 15 min of stirring after precipitation started. The resulting mixture was cooled to room temperature (RT), as shown in Scheme 1. The resultant compound was obtained through filtration, followed by washing with DD and drying at RT for 24 h.

### 2.3. Characterization and Instrumentation Analysis

The synthesized composite was characterized by different types of techniques, such as microscopic and spectroscopic.

#### 2.3.1. Analysis of Functional Group

The Fourier transform-infrared (FT-IR) spectroscopy was utilized to verify the chemical interaction between the guava leaf powder particles and metal oxides, to identify the functional groups within the resulting composite, and to assess the bonding interaction between the adsorbent and the contaminant. The FT-IR spectra were recorded using a Shimadzu spectrometer (Kyoto, Japan) in the mid-infrared region (400–4000 cm^−1^) with KBr pellets.

#### 2.3.2. Characterization of Crystal Size and Phase

The powder X-ray diffraction (XRD) pattern was demonstrated by an Ultima IV diffractometer (Rigaku, (Tokyo, Japan)) over a 2θ range of 10–80° to analyze the phase and crystal size of the composite.

#### 2.3.3. Morphology Analysis

To examine the surface morphology, particle shape, structures, and size of the prepared composite, two advanced microscopic techniques, such as scanning electron microscopy (SEM) and Transmittance electron microscopy (TEM), were utilized. SEM images were obtained using a Zeiss EVO-50 microscope operating at 20 kV (Jena, Germany), while a Tecnai F30 S-Twin microscope (Hillsboro, OR, USA) was utilized to capture the TEM images.

#### 2.3.4. Elemental Analysis

The elemental composition of the composite was examined through an Energy-Dispersive X-ray spectrometer (EDAX/EDX) [21]. The Zeiss EVO-18 (Jena, Germany) energy-dispersive X-ray spectroscope was utilized to conduct EDX analysis.

#### 2.3.5. Zero-Point Charge (ZPC)

The ZPC of the samples was conducted using the salt addition method. The ZPC of the adsorbent surface plays a critical role in regulating the transfer of the solute from the liquid phase to a solid phase, which is significantly influenced by the pH of the reaction medium. When the pH is adjusted by adding acids or bases, the surface acquires either a positive or a negative charge. Among the various methods available for determining ZPC, the salt addition method was chosen for the present composite due to its simplicity and accuracy [52].

Briefly, Erlenmeyer flasks (EFs, 100 mL) containing 0.01M KNO_3_ (aq) solution (50 mL) and a fixed amount of composite (200 mg) were prepared with initial pH value (pH_i_) between 2 and 10. The resultant mixtures were placed in a water bath shaker set to 180 rpm and maintained at room temperature for 24 h. After shaking, the final pH (pHf) of each mixture was recorded, and the change in pH (ΔpH = pH_i_ − pHf) was calculated. A plot of ΔpH versus pH_i_ was used to determine the ZPC of the composite. The pH measurements were conducted using a pH meter (Labtronics microprocessor, Model LT-50, Panchkula, Haryana, India).

### 2.4. Preparation of Stock Solution and Its Measurements

A stock solution of MB (1.0 g L^−1^) dye has been prepared using water (DD) and then diluted to the prescribed concentrations for the adsorption experimental studies. The MB dye concentration of the solution was directly measured at the λ_max_ of 660 nm using a UV-Vis spectrophotometer (T80-UV/VIS, PG Instruments Ltd., Leicestershire, UK). For constructing the calibration curves, the absorbance of the standard adsorbate solutions was measured at fixed wavelengths and varying concentrations. The resulting data plot demonstrated the high accuracy of the calibration curves, confirming the validity of the Beer-Lambert law within the given concentration range.

### 2.5. Adsorption Experiments

The adsorption efficiency of the synthesized composite was evaluated through batch mode adsorption experiments, agitating a series of EF of 100 mL containing a known adsorbent (*m*, grams) and MB solution (volume of MB dye solution = 10 mL with a specific initial concentration (*C_o_*, mg L^−1^)) at 27 °C, pH (7.0), and a mechanical shaker (180 rpm) for the required duration. To optimize the adsorption process, experiments were conducted under varying conditions such as time (15–120 min), pH (2–10), temperature (27–45 °C), MB concentration (10–45 mg L^−1^), or adsorbent dosage (0.5–3.0 g L^−1^) while keeping the remaining variables fixed. The adsorption results obtained were verified by thermodynamics, isotherm, and kinetic models. After each experimental run, the adsorbent-loaded material was extracted from the agitated solution, and the absorbance of the remaining adsorbate concentrations (*C_e_*) in the solutions was measured at 660 nm using the UV-visible spectrophotometer.

The equilibrium adsorption capacity (*Q_e_*, mg g^−1^) and removal efficiency (%) were determined using the equations below (Equations (1) and (2)) [52]:

(1) $$Equilibrium\;adsorption\;capacity\;(Q_{e})\;=\frac{\left(C_{o}-C_{e}\right)V}{m}$$

Here *V* represents the MB volume solution (litter), and all other constants are represented in detail in the above paragraph.

The MB removal efficacy (% adsorption) was calculated from the following equation [52]:

(2) $$Removal\;efficiency\;\left(\%\right)=\;\left(\frac{C_{o}-C_{e}}{C_{o}}\right)\times 100$$

All the above experiments were conducted in triplicate, and the average values were calculated for the corresponding parameters.

### 2.6. Thermodynamic Studies

To examine the effect of temperature and thermodynamic behavior on the MB adsorption of the synthesized nanocomposite from water, a series of adsorption tests was conducted at three distinct temperatures (27 °C (300 K), 35 °C (308 K), and 45 °C (318 K)). The amount of adsorbent used was fixed (2.0 g L^−1^) and the concentrations of the MB dye were systematically adjusted (10–45 mg L^−1^), and allowed to interact for a predetermined period (90 min) at 7.0 pH.

The thermodynamic variables such as Δ*G*° (free energy change (kJ mol^−1^)), Δ*H*° (enthalpy change, (kJ mol^−1^)), and Δ*S*° (entropy change kJ mol^−1^ K^−1^) were calculated to demonstrate the thermodynamics of MB adsorption [53]:

The ∆G° values were calculated using Equation (3).

(3) ∆G°=RTlnKc

Here, *Kc* = *Q_e_*/*C_e_*, *R* and *T* denote the gas constant and temperature (Kelvin), respectively. All other parameters retain their usual meaning.

The ΔH° and ΔS° values were derived from Equation (4).

(4) ∆G°=∆H°−T∆S°

The interception and slope of the plot drawn between Δ*G*° and *T* gave the values of the Δ*H*° and Δ*S*°, respectively.

The negative value of Δ*G*° refers to the feasibility and spontaneity of the adsorption process. In addition, the Δ*G*° values between 0 and −20 kJ mol^−1^ suggest that the uptake of the pollutant on the solid surface is governed predominantly by physisorption rather than chemical bonding [52,53]. The thermodynamic character of the adsorption process (including whether it is endothermic or exothermic) was inferred from the sign of the Δ*H*° (a negative sign refers to exothermic, while a positive sign indicates an endothermic adsorption process). The Δ*S*° indicates the randomness of the system. If the Δ*S*° is positive, the randomness increases, and if it is negative, the randomness decreases.

### 2.7. Isotherm Models

The type of interactivity between the adsorbent and the adsorbate in terms of the nature of the adsorbent surface can be demonstrated by fitting the obtained adsorption data to various models, such as Langmuir and Freundlich.

The Langmuir isotherm assumes that the adsorption occurs through the formation of a single, uniform layer of a solute species on the adsorbent surface, with each site acting independently and without interaction between neighboring adsorbed species [52].

The Langmuir isotherm parameters can be derived using linearized and nonlinear plots. The Langmuir isotherm Equations (5) and (6) are used to draw these plots (*C_e_*/*Q_e_* vs. *C_e_*), respectively.

(5) $$\frac{C_{e}}{Q_{e}}=\frac{C_{e}}{Q_{o}}+\frac{1}{Q_{o}b}$$

(6) $$Q_{e}=\frac{Q_{o}bC_{e}}{1+bC_{e}}$$

where *Qo* is the maximum adsorption capacity, representing the monolayer Langmuir capacity, and b is the isotherm coefficient, representing the adsorption intensity.

The separation factor, RL, another Langmuir constant that can be used to predict the affinity of pollutants with solid surfaces, is a dimensionless constant. This factor is an important characteristic of the equilibrium state in adsorption, and it is calculated by using the following relationship (Equation (7)) [51,54]:

(7) $$R_{L}\;=\frac{1}{\left(1+bC_{e}\right)}$$

The separation factor value defines the adsorption process as reversible, irreversible, or linear. If *R_L_* = 0 represents the irreversible adsorption process, and 0 < *R_L_* < 1 is indicative of a favorable sorption reaction. The R_L_ > 1 represents a non-favorable sorption reaction, and R_L_ = 1 represents the linear relationship.

In contrast, the Freundlich isotherm accounts for the adsorption on surfaces with non-uniform energy distribution, where solute molecules or ions can accumulate in multiple layers [52].

The Freundlich isotherm parameters can be derived using Equations (8) and (9), which give linear and nonlinear plots (*lnQe* vs. *lnCe*), respectively.

(8) $$\log Q_{e}=\log k_{F}+\frac{1}{n}\log C_{e}$$

(9) $$Q_{e}=k{FC_{e}}^{1/n}$$

Here *kF* is the Freundlich adsorption capacity, and n represents the heterogeneity factor. If the n value falls between 1 and 10, it indicates heterogeneous adsorption behavior.

R^2^ is the regression coefficient, which represents the best fit of the linear plots. For nonlinear plots, the error function is used; a lower ARE value indicates the best fit [54].

### 2.8. Adsorption Kinetics

The adsorption rate of a system is influenced by multiple factors, including the characteristics of the adsorbent surface, i.e., either homogeneous or heterogeneous, the adsorbate structure found in the solution media; and the interaction pattern between the adsorbate and adsorbent. Therefore, the whole adsorption process is governed by these steps [52]. The general steps involved in the adsorption process, which decide the rate-determining step, are mass transfer, film diffusion (FD), and intraparticle diffusion (IPD) [54].

Understanding the rate and underlying mechanism of adsorption is essential for the effective design of a water purification method. Adsorption kinetics and the associated rate-limiting steps provide insight into how the adsorbate transfers from the liquid phase to the solid surface, and these mechanisms can be elucidated by fitting time-dependent sorption data to the appropriate kinetic models. The famous and common models are pseudo-first order (PFO), pseudo-second order (PSO), Elovich, and Weber-Morris kinetic models, which are employed [54].

The PFO kinetic model was originally proposed by Lagergren and later refined by Ho, and it assumes that the adsorption rate is proportional to the number of unoccupied active sites on the adsorbent at equilibrium [52].

The PFO kinetic parameters can be derived using Equation (10), which gives a linear plot (*log*(*Q_e_* − *Q_t_*) vs. *t*).

(10) $$\log(Q_{e}-Q_{t})=\log Q_{e}-\frac{k_{1}}{2.303}t$$

In contrast, the PSO model suggests that the adsorption behavior of a solid–liquid system depends on both the properties of the aqueous phase and the characteristics of the adsorbent surface [52]. The model further implies that the adsorption rate is related to the square of the difference between occupied and available sites at equilibrium [51]. The PSO model is particularly suitable for systems in which the adsorption is governed by a chemical interaction between the adsorbate and the solid surface [52]. The PSO kinetic parameters can be derived using Equation (11), which gives linear plot (t/*Q_t_*) vs. *t*).

(11) $$\frac{t}{Q_{t}}=\frac{1}{h}+\frac{t}{Q_{e}}$$

The Elovich kinetic model has been widely applied to describe the solid–liquid adsorption processes dominated by the chemical interactions, particularly on the energetically heterogeneous solid surfaces [52]. This model provides insight into the surface coverage evolution as well as adsorption (α) and desorption (β) rate, and it also reflects the activation energy associated with the chemisorption process [52]. These parameters can be derived using Equation (12), which gives a linear plot (*Q_t_* vs. *ln*(*t*)).

(12) $$Q_{t}=\frac{1}{\beta }\ln(\alpha \beta )+\frac{1}{\beta }\ln t$$

A comparison of the coefficients of determination (R^2^) and the associated parameters derived from these models allows the assessment of their suitability in fitting the time-dependent adsorption data.

The rate-controlling steps of the solid–liquid adsorption systems involve three sequential steps, viz., surface adsorption, particle diffusion, and thin boundary layer formation, which can be described by the mechanistic model such as Weber-Morris (WM) [54].

When adsorbate molecules or ions migrate into the internal pores of an adsorbent and subsequently bind to the surface through chemical or physical interactions, the WM model is often used to describe the time-dependent adsorption behavior [54]. The WM model, which is a combination of particle diffusion, surface adsorption, and thin boundary layer formation models, can define the rate-controlling step associated with the solid–liquid sorption systems [54]. When the WM plot is straight with a good regression coefficient and passes through the origin, then only the intraparticle diffusion (IPD) step will control the whole process; otherwise, the IPD as well as the film diffusion (FD) are considered as rate-deciding steps [54].

The WM model can be represented by Equation (13).

(13) $$Q_{t}=k_{ipd}t^{0.5}+C$$

Here, the k_ipd_ is a rate constant, C represents boundary layer thickness, and t^0.5^ represents the half-life time.

The value of k_ipd_ and C can be derived from the slope and intercept of the WM plot between *Q*_t_ and t^0.5^, respectively. Generally, the WM plot is a multiline plot in which each line represents an adsorption stage (step). The first line represents the FD step, while the second line refers to the IPD step. A lower value of slope (k_ipd_) and a higher value of intercept (C) for a line (stage) indicate a slower adsorption rate on that stage, which is the rate-determining step.

### 2.9. Recyclability of Exhausted Adsorbents

It is essential to separate, regenerate, and reuse the exhausted adsorbents to make the water treatment process more affordable and reliable [52]. This can be accomplished by firstly separating the adsorbate-loaded adsorbents from water, followed by desorbing the adsorbate surface by adjusting the pH of the cleaning-water system using acidic and/or alkaline solutions [51].

In this study, to detach (regeneration of the adsorbent via desorption) MB from the MnFe_2_O_4_/GL surface, 0.1 mole of HCl solution (50 mL) was added to a 100 mL Erlenmeyer flask containing a 1.0 g amount of the respective MB-loaded MnFe_2_O_4_/GL and then shaken for 4 h at room temperature at 180 rpm. After shaking, the regenerated adsorbents were separated using the Wattman filter paper No. 1, Sigma Aldrich, St. Louis, MI, USA washed with DD to neutralize their pH, then dried, and reused for another adsorption–desorption cycle. This process was further repeated five times to trace out the appropriate reusability of the MnFe_2_O_4_/GL for further adsorption.

## 3. Result and Discussion

The prepared composite was analyzed using various characterization techniques to examine its morphology, functionality, and physicochemical properties.

### 3.1. Characterization

**Figure 1:**
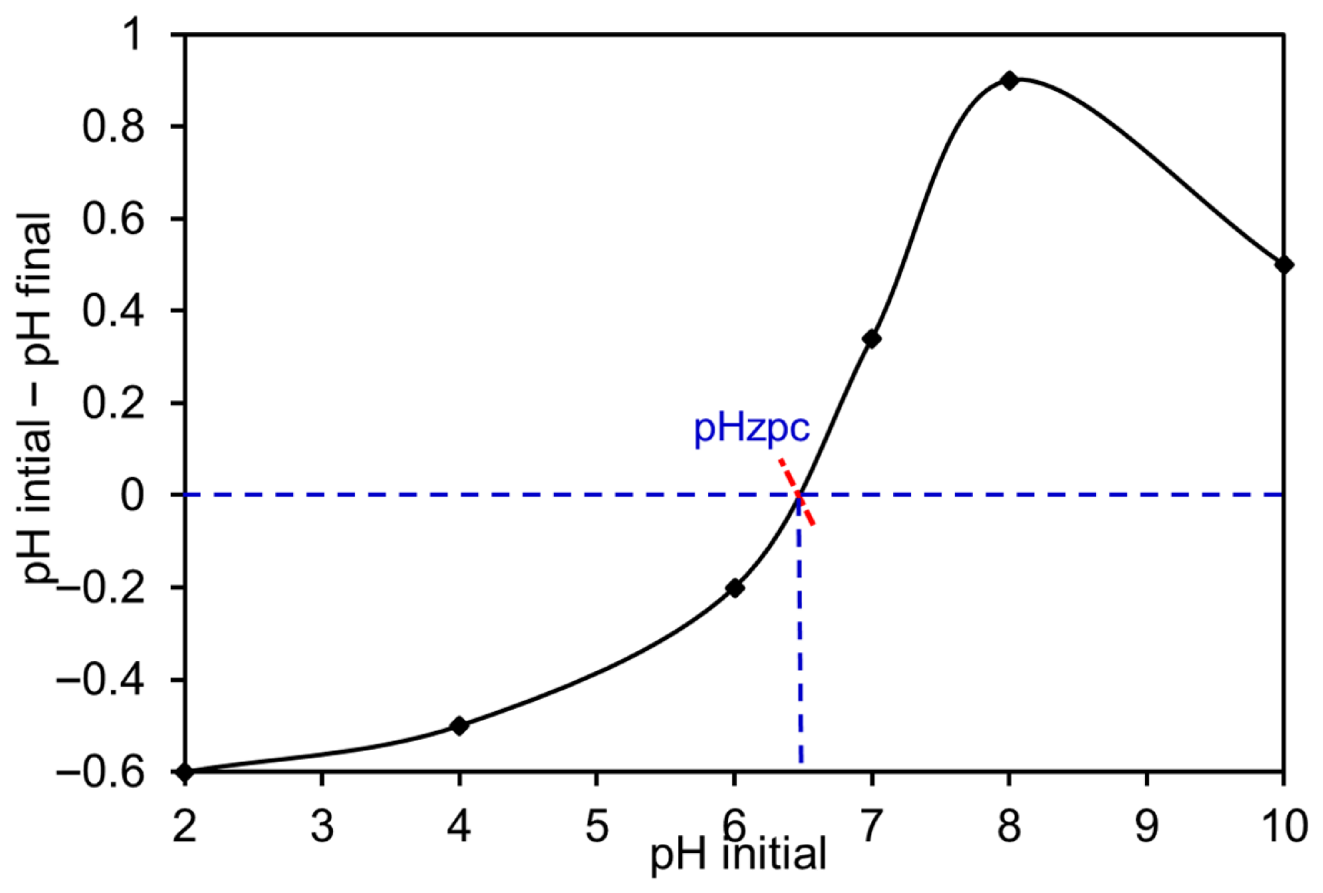
Plot of pH_p_zc for MnFe_2_O_4_/GL adsorbent.

The MnFe_2_O_4_/GL adsorbent exhibited a pH_p_zc of approximately 6.5 (Figure 1), measured using the salt addition procedure as described in Section 2.3.5.

At this pH, the net charge on the adsorbent surface is zero. At pHs lower than this point, the functional groups on the adsorbent surface protonate, creating a positive charge on the adsorbent surface. At pHs higher than this point, the adsorbent deprotonates, creating a negative charge on the adsorbent surface. These adjustments in the surface charge of the adsorbent through pH changes help in the deposition of the dye on the adsorbent surface [52].

**Figure 2:**
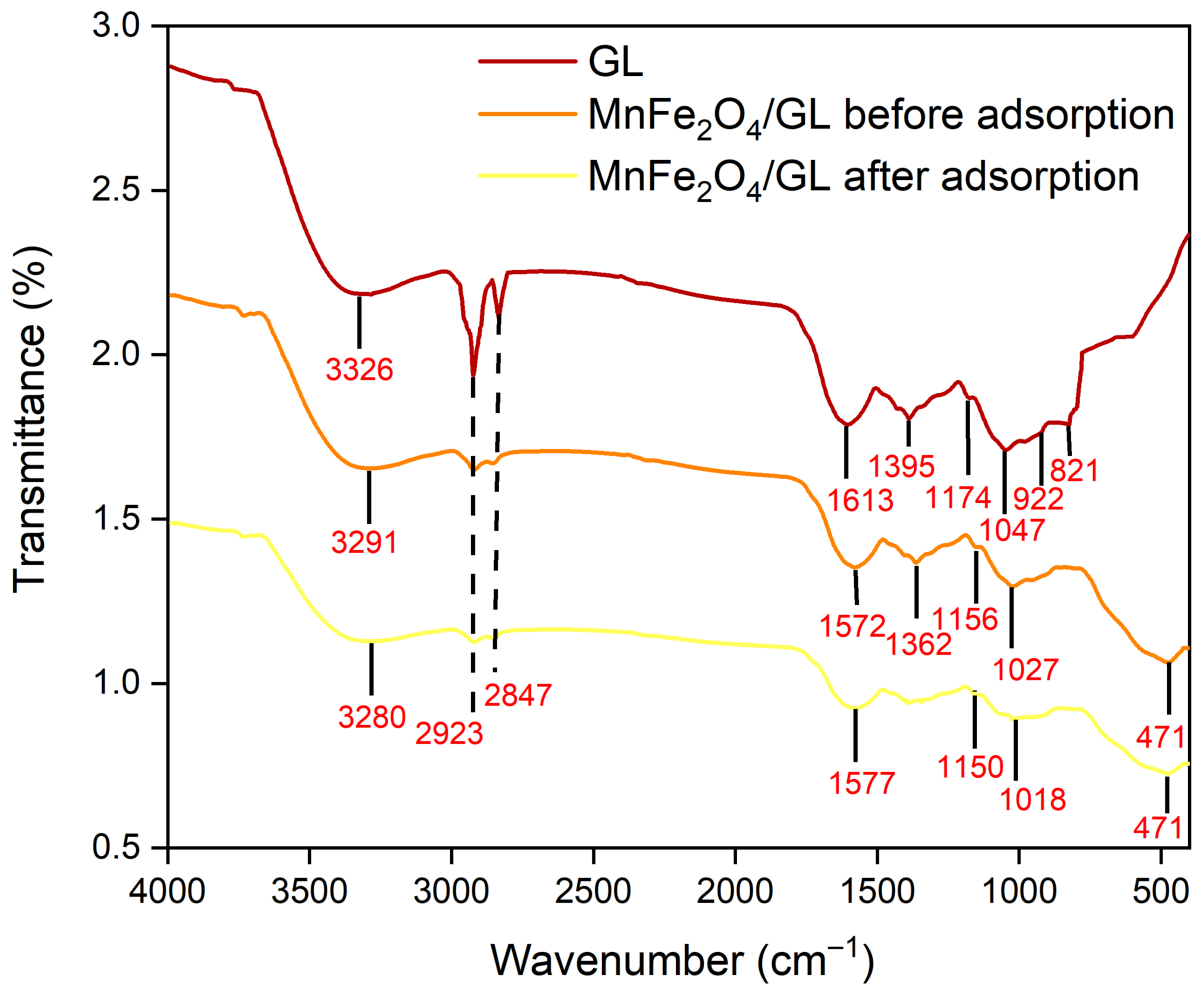
FTIR of GL powder, MnFe_2_O_4_/GL before adsorption and after adsorption.

The presence of various functional groups in the composite can be understood by the FT-IR analysis. For the current study, the IR spectra of the prepared sample of MnFe_2_O_4_/GL composite (Figure 2) were compared with the virgin GL (Figure 2). Compared to the FT-IR spectrum of GL, the FT-IR spectrum of the composite sample exhibited characteristics of absorption peaks belonging to the GL framework functional groups [43], as well as metal-oxygen MnFe_2_O_4_ NPs, as presented in Table 1 [51].

In the spectrum of GL (Table 1), the assigned bands were for -OH stretching, -C-H stretching, N-H bend/amide I, C-O stretching, H-C-O stretching, C-O-C stretching, and/or C-O stretching, C-O, C-C stretching/bending, and out-of-plane -C-H bending.

The IR spectrum of the composite revealed the same peaks observed in the spectrum of the GL; however, some shifts in peak positions were noted, while certain peaks disappeared entirely, as illustrated in Table 1. The primary cause for these shifts and disappearances lies in the interactions occurring between the GL and the NPs. For instance, the hydroxyl peak shifted from 3326 cm^−1^ to 3291 cm^−1^, indicating the formation of hydrogen bonds between the NP-OH and the GL-OH. Similarly, the shifts were observed in the peaks corresponding to N-H bending/amide I (1613 to 1572 cm^−1^), C-O stretching (from 1395 cm^−1^ to 1362 cm^−1^), and H-C-O stretching, C-O-C stretching, and/or C-O stretching as detailed in Table 1, resulting from electrostatic interactions between the NPs and the GL functional groups. Furthermore, the peak originally located at 950–800 cm^−1^ vanished completely, signifying strong interactions between the NPs and the GL functional groups. There was no change in the C–H stretching frequency of the GL leaf, indicating the specific interaction between the NPs and the GL surface. In addition to these organic peaks, one distinct peak was detected in the composite spectrum at 471 cm^−1^, which primarily corresponds to the metal-oxygen bonds. This comparative IR study confirms the existence of interactions between the NPs and the guava leaf, leading to the formation of a composite material characterized by the presence of various functional groups. These functional groups serve as active sites.

The synthesized composite material was subsequently utilized for adsorption experiments, and a post-adsorption IR analysis was also conducted. Various shifts in peak positions were also discernible in the post-adsorption IR spectrum, primarily reflecting the interactions occurring between the MB dye and the composite material. For example, shifts were observed in the H-C-O stretching, C-O-C stretching, and/or C-O stretching (1156 cm^−1^ to 1150 cm^−1^ and 1027 cm^−1^ to 1018 cm^−1^), N-H bend/amide (from 1572 cm^−1^ to 1577 cm^−1^), and hydroxyl peaks (from 3291 cm^−1^ to 3280 cm^−1^), which predominantly indicate the occurrence of both electrostatic and hydrogen-bonding interactions with the MB dye, respectively. Consequently, it can be concluded that the adsorption of MB onto the surface of the adsorbent was driven by a combination of electrostatic bonds and non-electrostatic bonds.

**Figure 3:**
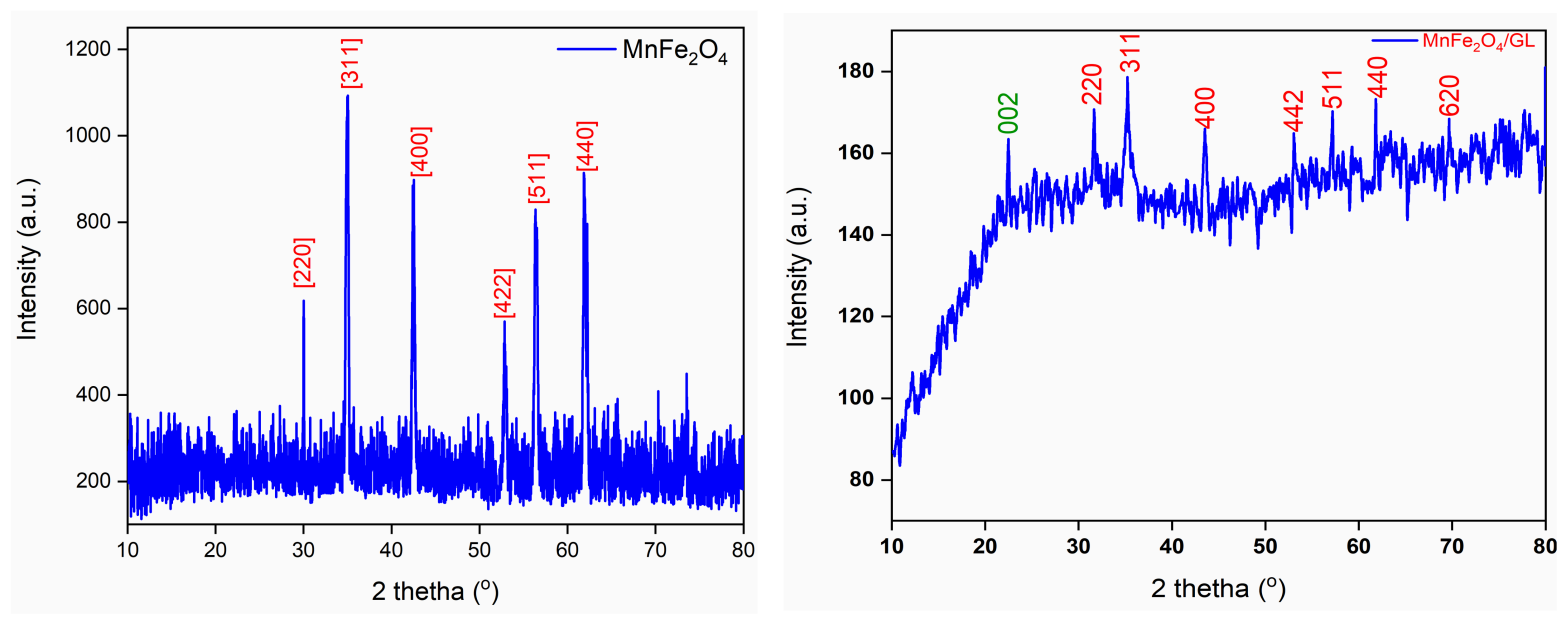
XRD pattern of MnFe_2_O_4_ and MnFe_2_O_4_/GL.

The diffraction (X-ray) pattern of both bare MnFe_2_O_4_ (reference material) and MnFe_2_O_4_/GL (Figure 3) exhibited the characteristic XRD peaks at specific 2θ values ~31°, ~35°, ~43°, ~53°, ~56°, ~61°, and ~70°, belonging to the plane of [220], [311], [400], [422], [511], and [440] [56,57]. In the XRD of MnFe_2_O_4_/GL, the peak at ~22° was assigned to the corresponding plane [002] suggests the presence of a carbon framework (GL) in the composite [52]. These peaks and their corresponding planes confirm the presence of the MnFe_2_O_4_ crystal in the GL framework, as identified by matching the JCPDS File No. 73-1964 [56]. The XRD spectrum of MnFe_2_O_4_/GL aligns well with the previous research findings [51,56,57].

**Figure 4:**
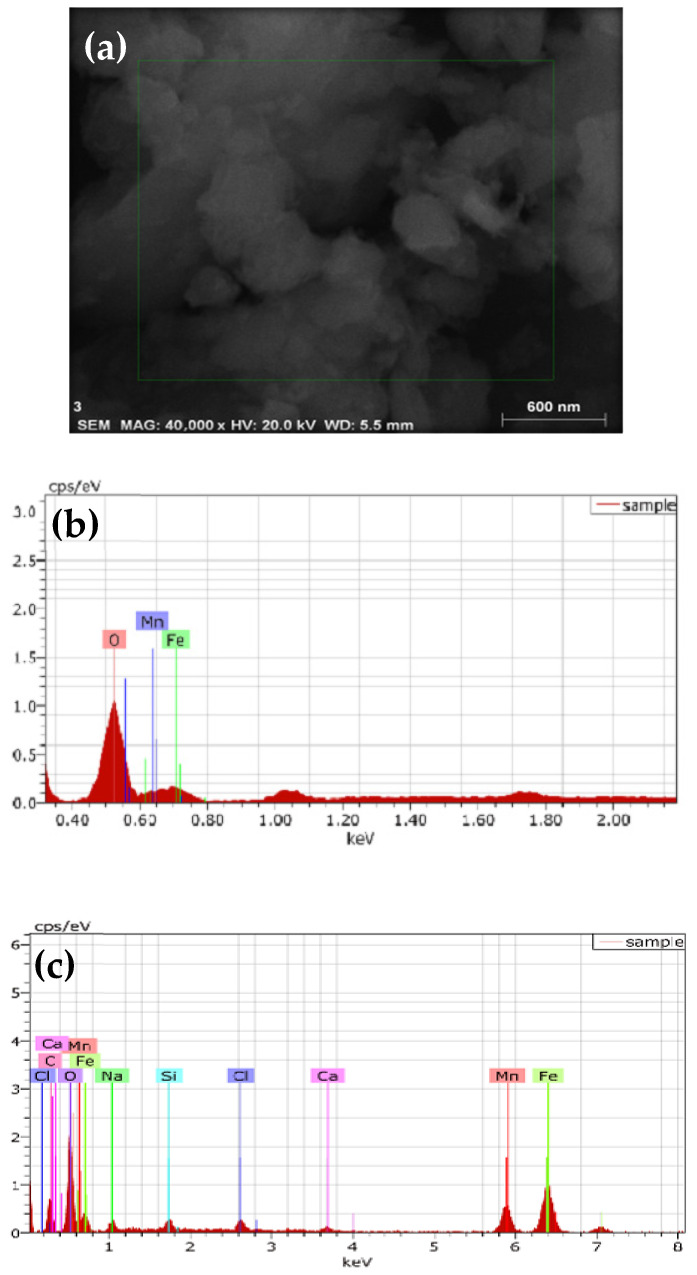
(**a**) SEM image of MnFe_2_O_4_/GL, (**b**) EDX spectra of MnFe_2_O_4_, and (**c**) EDX spectra of MnFe_2_O_4_/GL.

The SEM image of MnFe_2_O_4_/GL (Figure 4a) showed the growth of irregularly shaped MnFe_2_O_4_ NPs in the GL framework. The comparative elemental analysis of MnFe_2_O_4_ (Figure 4b) and MnFe_2_O_4_/GL (Figure 4c) through EDX spectra showed that C, N, O, Fe, and Mn were present in the composite, with some other impurities in MnFe_2_O_4_/GL (Figure 4c). The detected impurities include Na, Cl, Ca, and Si. The presence of Na and Cl may be related to the synthesis precursors (FeCl_3_ and NaOH), while Si and Ca are probably due to intrinsic mineral impurities present in the GL used during preparation.

**Figure 5:**
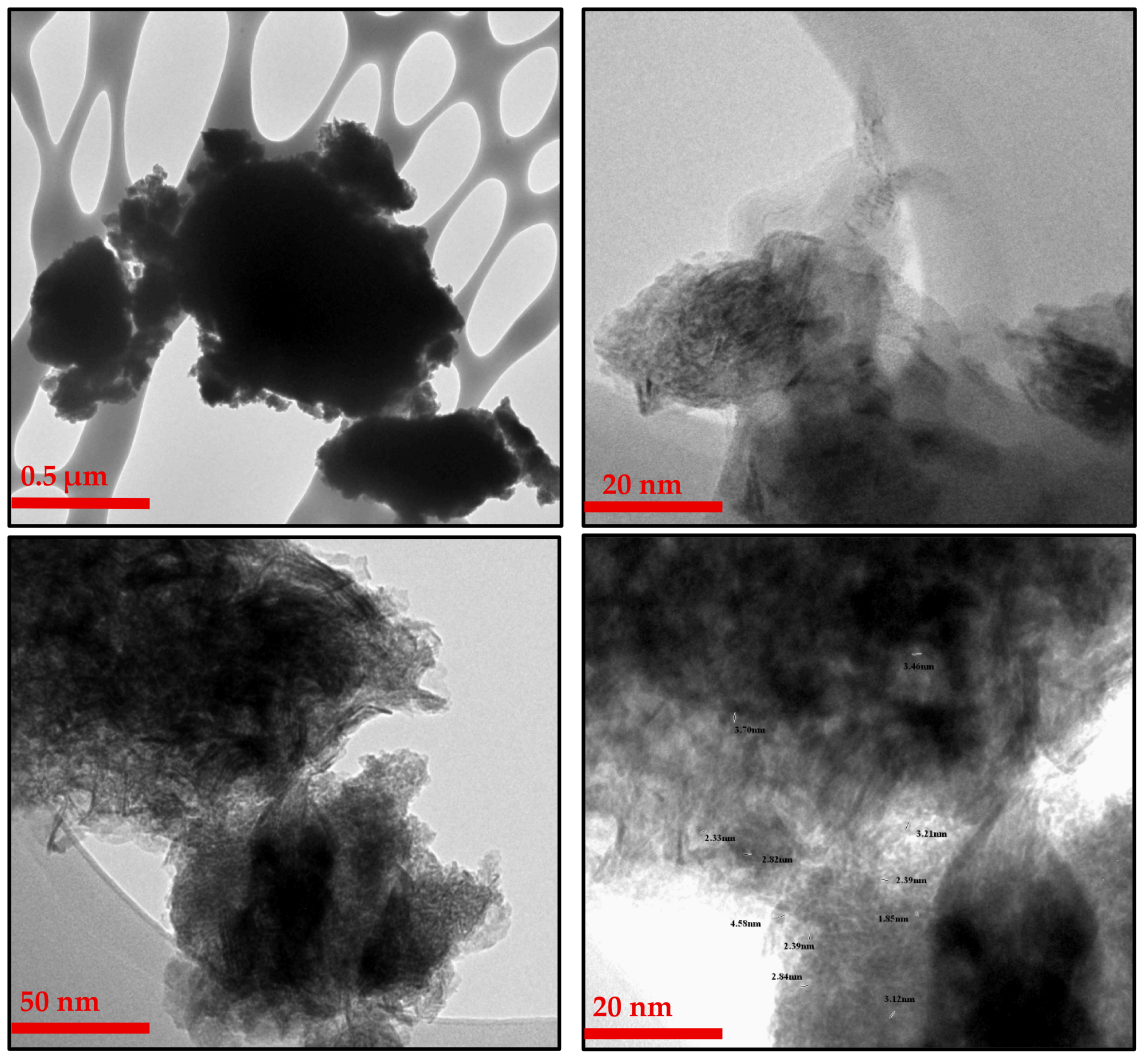
TEM image of MnFe_2_O_4_/GL at various magnifications.

Here, TEM images are shown at different magnifications (Figure 5). In the TEM images, it can be seen how the NPs of MnFe_2_O_4_ are trapped in the organic framework. In the first TEM image, numerous vacuoles can be clearly seen, which are actually plant cells in the GL. It is natural to find numerous functional groups in the GL’s vacuoles and cell walls (cellulosic surface), which may have facilitated the adhesion of NPs (through electrostatic and non-electrostatic interactions) to the GL surface [52]. Therefore, MnFe_2_O_4_ NPs, with a size range of 3–5 nm, are evenly distributed on the GL surface (which contains vacuoles). The aggregation (due to the non-electrostatic interactions between the NPs) can also be seen in the TEM images.

From the current physico-chemical analysis, it can be concluded that the prepared NC contains many functional groups, which can prove to be very beneficial for the adsorption process. This composite was also investigated for an adsorption application.

### 3.2. Adsorption Studies

The adsorption performance of MnFe_2_O_4_/GL was investigated for MB in batch mode as follows:

#### 3.2.1. Effects of Adsorbent Dosage, Concentration, Temperature, and pH

**Figure 6:**
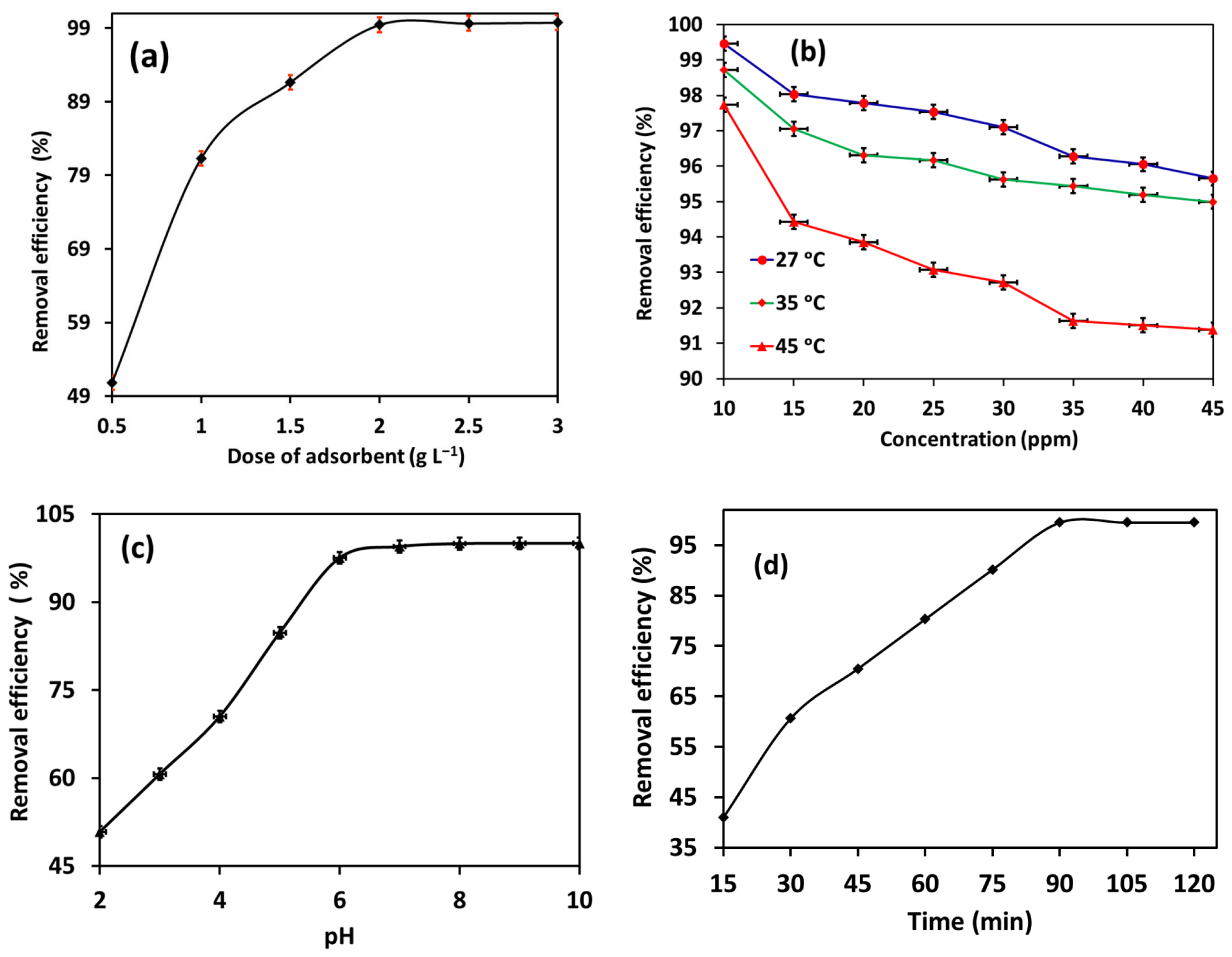
Effect of (**a**) adsorbent dose, (**b**) combined effect of temperature and initial concentration of MB solution, (**c**) solution pH, and (**d**) contact time onto the MB adsorption.

The adsorption efficiency of MnFe_2_O_4_/GL was examined at the absorbent dosage from 0.5 to 3.0 g L^−1^, as shown in Figure 6a. A MnFe_2_O_4_/GL (2.0 g L^−1^) was adequate to remove more than 50% MB from a 10 mg L^−1^ solution at 0.5 g L^−1^. The removal efficiency (%) of MnFe_2_O_4_/GL for MB was evaluated with increasing dosage. The removal efficiency (%) can be seen to increase with increasing adsorbent dosage (Figure 6a). This is primarily because as the adsorption dosage increases, the active sites on the adsorbent’s surface also increase, allowing it to adsorb a larger amount of dye. It can also be seen in the current study that the adsorbent exhibited very good removal efficiency (%), so it reached equilibrium very quickly. For this study, at 2.0 g L^−1^, adsorption reached equilibrium, where it removed almost 99% of the MB dye from the water [51].

This study also investigated the effect of dye concentration on the adsorption performance of MnFe_2_O_4_/GL (Figure 6b). The dye concentration was increased from 10 mg L^−1^ to 45 mg L^−1^, while other conditions, such as adsorbent dose (2.0 g L^−1^), time (120 min), pH (7.0), and shaking speed (180 rpm), were kept constant. The results showed that for a fixed dose, increasing concentration increased the adsorption capacity, while decreasing the removal efficiency (%). The main reason for the increase in adsorption capacity can also be understood from the adsorption capacity formula (Equation (2)), which states that the adsorption capacity of an adsorbent is directly proportional to the dye concentration in the solution; therefore, the adsorption capacity increases with increasing concentration. It increases until every active site of the adsorbent is exhausted, after which the adsorption capacity reaches equilibrium. In the current experiment, the adsorption capacity of MnFe_2_O_4_/GL (with 2.0 g L^−1^) increased from approximately 5.0 to 21.0 mg g^−1^ as the MB concentration in solution rose from 10 to 45 mg L^−1^ at 27 °C (Figure 6b) [52].

Conversely, the main reason for the decrease in the removal efficiency (%) is that at a fixed dose, the number of active sites on the adsorbent surface remains fixed, and as the dye concentration increases, only a limited amount of dye can be absorbed by the fixed sites, leading to a decrease in the overall removal efficiency (%) at high concentrations.

This effect of the concentration was also observed at different temperatures (27–45 °C) (Figure 6b). It was further found that as the temperature increased, the removal efficiency (%) decreased at each concentration. The decrease in removal efficiency (%) with increasing temperature indicates an exothermic process, confirming that as the temperature increases, the bond formed between the adsorbate and the adsorbent, which is slightly weaker, breaks at higher temperatures and releases energy. Furthermore, due to increased randomness at higher temperatures, the adsorbate is unable to occupy specific adsorption sites, leading to decreased adsorption at higher temperatures [51,52].

A detailed investigation of the pH effect was also conducted in this study. To demonstrate this effect, adsorption experiments were performed at optimal conditions with pH ranging from 2 to 10. The result is shown in Figure 6c. The result indicates that removal efficiency increases with increasing pH. That is, at acidic pH, the removal efficiency (%) for the MB dye was very low (~50% at pH 2), while at alkaline pH the removal efficiency (%) was significantly higher (~100% at pH 10). However, it can be seen that above pH 7, the removal efficiency (%) remained almost at equilibrium. To further understand the pH effect, a comparative study of the adsorption capacity was conducted, and it was found that as the pH increases, the adsorption capacity increased, from 2.5 mg g^−1^ to 5.0 mg g^−1^. This variation in the adsorption capacity of the current adsorbent for the MB dye at different pHs can be explained by the fact that at acidic pH, the adsorbent surface is cationic, meaning that below 7 pH, the adsorbent surface undergoes protonation, becoming positively charged. MB, a cationic dye, exhibits repulsion by the positive surface, resulting in a significant decrease in the adsorption capacity. Consequently, as the pH increases, deprotonation occurs at the surface, and the concentration of OH^−^ increases, causing the surface to become negative. This negative surface exhibits attraction to cationic dyes like MB, and thus, the adsorption capacity increases at higher pHs. This result can also be justified by pHzpc for the adsorbent, which came to 6.5, that is, below this pH, protonation occurs at an adsorbent surface, whereas above it, deprotection occurs, and due to these, the adsorption capacity fluctuates as mentioned above [52].

Notably, there were many functional groups on the surface, mainly oxygenous groups (hydroxyl, carboxyl, and carbonyl groups), as mentioned in the FT-IR analysis. These oxygenous groups are also capable of forming hydrogen bonds (non-electrostatic interaction) with MB. This is the reason that a significant amount was absorbed even at acidic pHs. Similar pH studies were observed in our previous research [43,44].

#### 3.2.2. Thermodynamics and Isotherms

**Figure 7:**
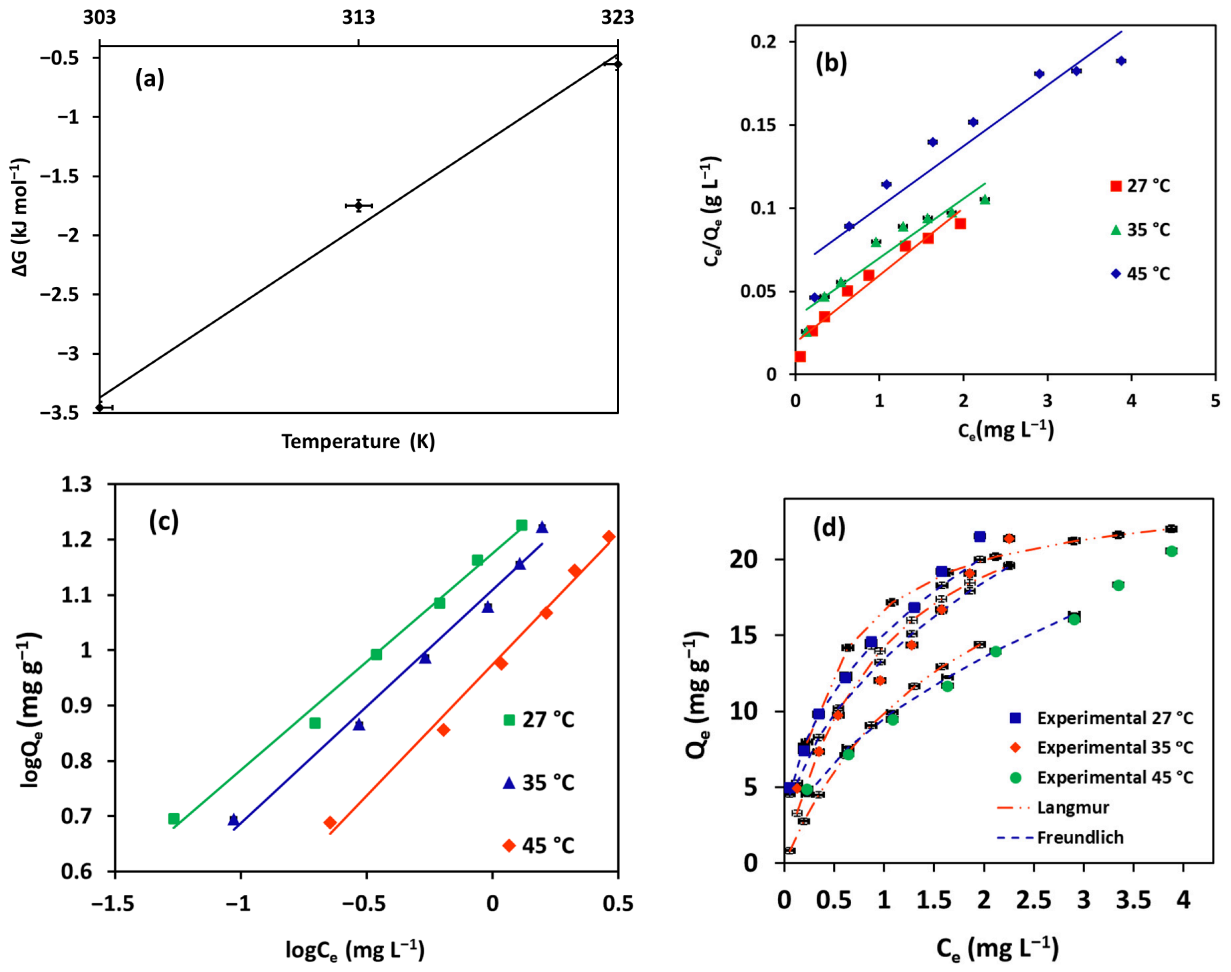
(**a**) Thermodynamics plot, (**b**) Langmuir plots, (**c**) Freundlich plots, and (**d**) isotherm simulation plot for MB adsorption.

From Figure 6b, it is evident that the removal efficiency of MnFe_2_O_4_/GL decreases at higher temperatures (45 °C) compared to lower temperatures (27 °C), indicating that the adsorption method is exothermic. This is further supported by the negative ΔH°, which further confirms the exothermic nature of the adsorption (Table 2) [53]. The negative ∆G°, calculated from the Gibbs equation, implies that the reaction is both feasible and spontaneous across all temperatures (Figure 7a). Additionally, the negative ΔS° value suggests a reduction in uncertainty (Table 2) [53].

The isotherm parameters have also been calculated, and their results are given in Table 3. The Langmuir adsorption capacities reduce with the rising temperature, further demonstrating that the adsorption process is exothermic in nature. The Langmuir constant (b) values have suggested the high affinity of MB adsorption at 27 °C (Figure 7b).

The *n* values from the Freundlich model, ranging from 1 to 10, represent the MB favorably adsorbed onto the heterogeneous surface. The lower k_F_ value was found to be at 45 °C, again suggesting the exothermic process and high affinity of MB adsorption at 27 °C (Figure 7c) [52].

The Freundlich isotherm plot showed good linearity with higher R^2^ values compared to the Langmuir isotherm. The fitting of the Freundlich isotherm demonstrates the multilayer adsorption of MB onto the heterogeneous surface of MnFe_2_O_4_/GL [52].

The non-linear isotherm simulation plots also confirm the fitting of the Freundlich model at all the investigated temperatures. Through the simulation plot, it can be observed that the experimental adsorption data exhibits a high degree of similarity with the data obtained from the Freundlich model, thereby demonstrating a low error (ARE) function [54].

#### 3.2.3. Adsorption Kinetics and Mechanism

The time-dependent adsorption profile (Figure 6d) indicated that within 60 min, 2.0 g of MnFe_2_O_4_/GL removed approximately 80% of MB from the 10 mg L^−1^ solution, with complete removal (~99%) after 90 min. This result is consistent with findings reported in previous studies. In brief, the adsorbent initially has a large number of free adsorbent sites, which, upon mass transfer, are easily filled by the adsorbate molecules and become saturated. As time passes a limited number of free adsorbent sites remain available; thus, incoming adsorbate molecules need specific pathways to adsorb onto the adsorbent surface. This phenomenon addresses the slow rate of adsorption as time increases. To gain further insight into the adsorption rate, the experimental time-dependent data were analyzed using kinetics models, and the calculated parameters are presented in Table 4 [54].

**Figure 8:**
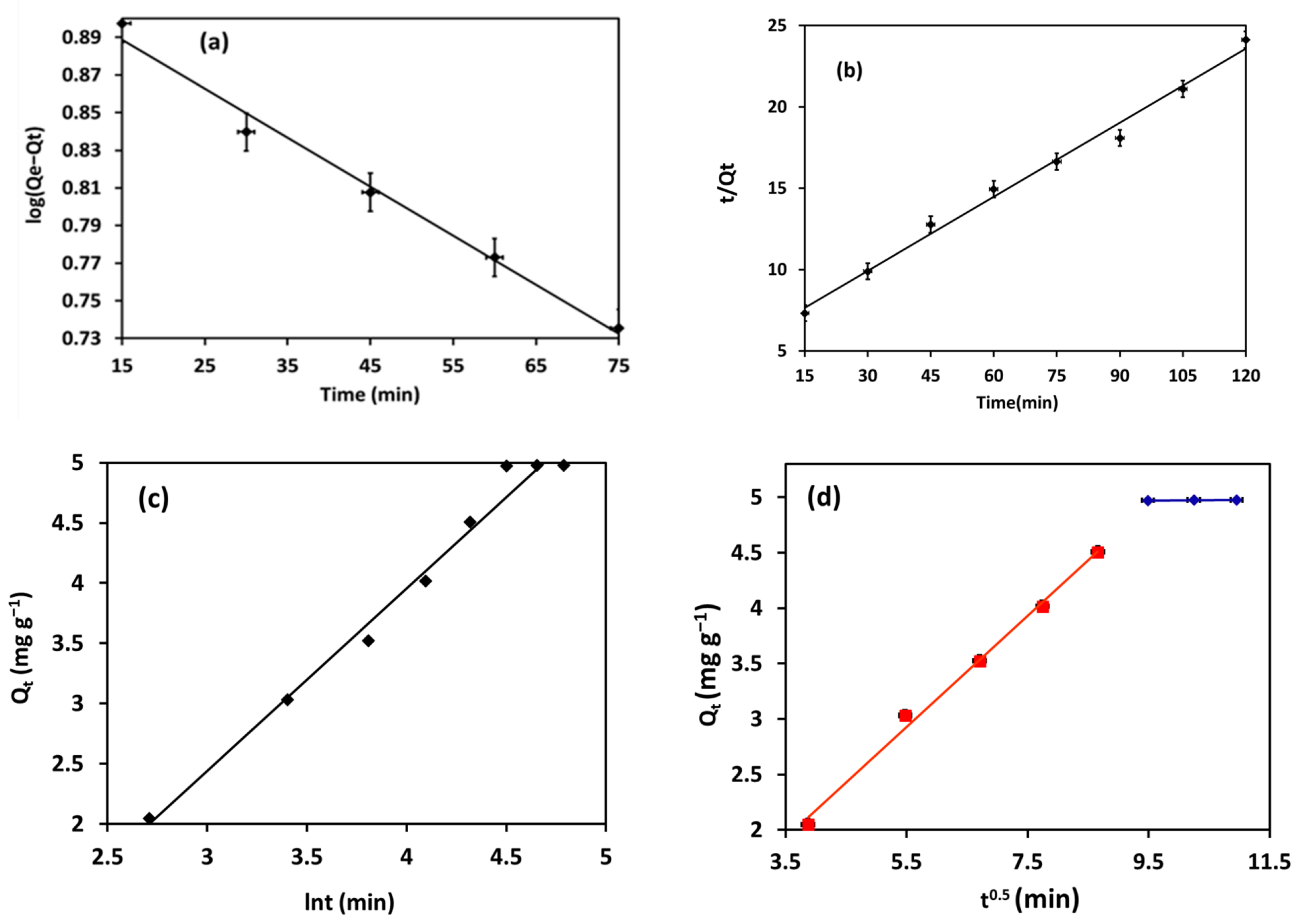
Kinetics plots (**a**) PFO, (**b**) PSO, (**c**) Elovich, and (**d**) WM plot.

The calculated coefficients of determination (R^2^) for both the PFO and PSO models (Figure 8a,b) were found to be close to unity (0.98). However, the experimental adsorption capacity matched the value calculated for the PSO model (Table 4). Therefore, the MB removal kinetics followed the PSO model more closely.

The greater value of α (6.07 × mg g^−1^ min^−1^) compared to β (0.66 g mg^−1^) determined using the Elovich model (Figure 8) suggested a lower desorption rate than adsorption rate (Table 5) [54]. The WM plot (Figure 8d), which was constructed between *Q*_t_ and t^0.5^, displayed two straight lines, indicating the involvement of two distinct stages in the MB adsorption process [54]. The kd and C (intercept) corresponding to the two adsorption stages, kd_1_, C_1_ for the FD stage, and kd_1_, C_2_ for the IPD stage, have been obtained from the WM plot and are listed in Table 5. The higher C value and lower kd associated with the second linear segment (red line) indicate the reduced MB uptake rate in the later stage. This suggests that the IPD governs the overall adsorption rate of MB. All of these adsorption experimental results are consistent with the explanations provided in previous research [54] for each corresponding stage.

#### 3.2.4. Results of the Recyclability of Exhausted Adsorbents

**Figure 9:**
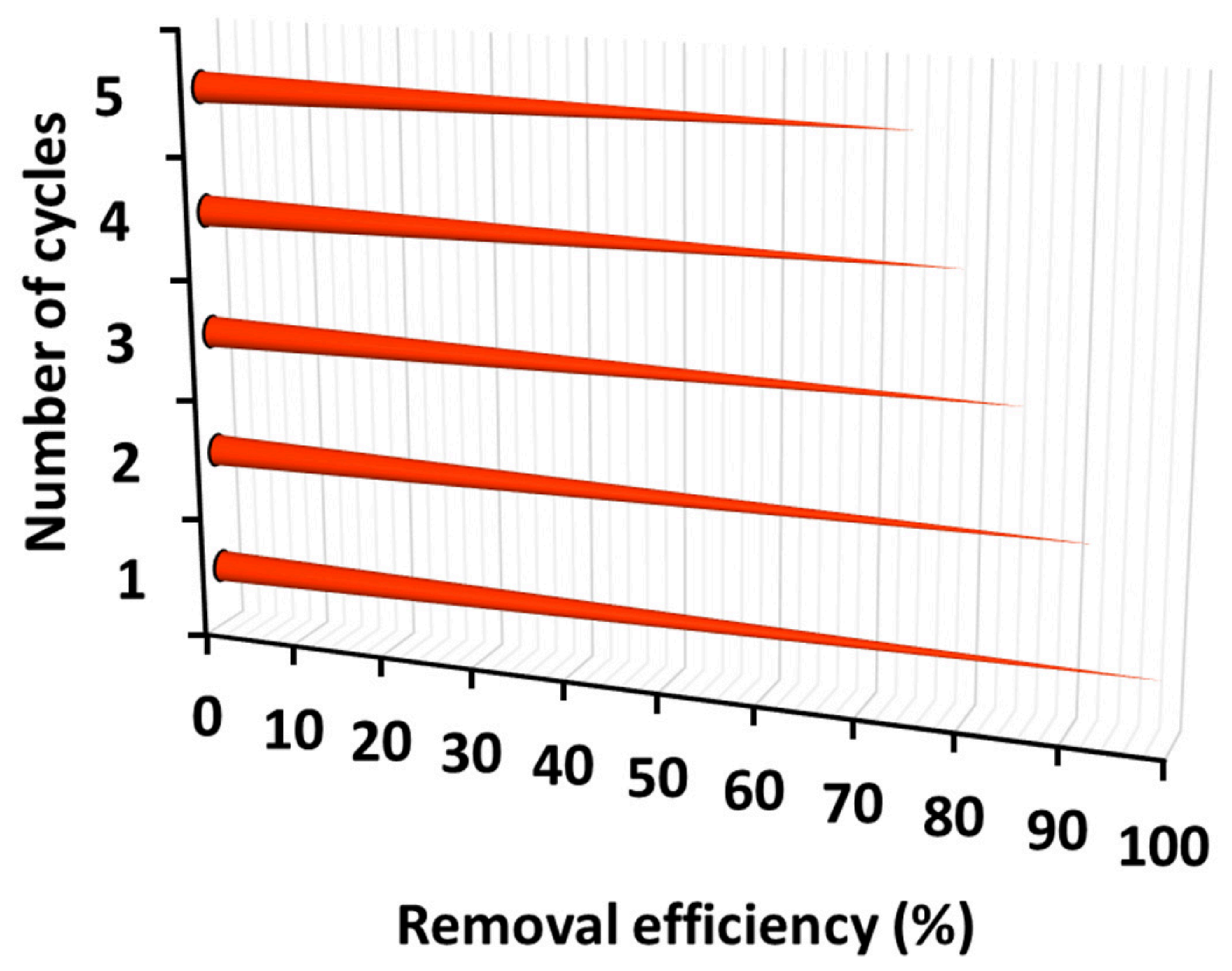
Regeneration and reusability result for MB adsorption.

Regeneration and reusability are essential for any adsorption study which defines the stability and sustainability of the adsorbent. The regeneration and reusability capacity of MnFe_2_O_4_/GL was also determined in this study. For this, HCl (50 mL of 0.1 M) was used as a regenerating agent. The degenerated adsorbent was used for five adsorption–desorption cycles, and the obtained result is shown in Figure 9. The reusability test results confirmed the effectiveness of the adsorbent for up to five cycles. There was only a nominal decline in the adsorption capacity in the first three cycles, which shows very good capacity for the current adsorbent. Only a 24% capacity reduction was found after the fifth cycle. This suggests the high stability and regeneration power of the MnFe_2_O_4_/GL.

## 4. Performance Evaluation of MnFe_2_O_4_/GL for MB Dye

In most studies involving comparative analyses, the Langmuir adsorption capacity is utilized to benchmark a specific adsorbent against others. However, relying solely on adsorption capacity for comparative studies is often considered a biased approach, as adsorption capacity is also contingent upon factors such as water pH, contact time, the inherent characteristics of the adsorbent, concentration, etc. Since the adsorption process in the current study did not strictly adhere to the Langmuir isotherm model, conducting a comparative analysis based solely on the Langmuir isotherm would not yield meaningful results. Consequently, to mitigate bias and enhance the effectiveness of the comparison, this study also calculated a Partition Coefficient (PC) [58]. The PC represents the ratio of the concentration of the adsorbate adsorbed onto the solid surface and the final concentration present in the water (Equations (14) and (15)), thereby serving as a less biased comparative metric.

(14) $$PC=Q_{e}/C_{e}$$

(15) $$PC=Q_{e}/{(C}_{o}\times Q_{t})$$

For the purpose of comparative analysis in this study, the PC for MB dye adsorption was calculated across various concentrations (Table 6a). It was observed that as the concentration increased, both the PC and the equilibrium adsorption capacity demonstrated a corresponding increase. When comparing the performance of the adsorbent utilized in this study against previously reported similar kinds of other adsorbents, a range of comparative PC values was observed, as illustrated in Table 6b. The PC value and adsorption equilibrium capacity (Q_e_) for the current adsorbent were found to be higher in comparison to the previously reported adsorbents. All these comparative observations were conducted under identical experimental conditions, specifically at a concentration of 10 mg L^−1^ and a pH of 7. In the present study, GL was utilized as a supportive material for composite preparation. Since GL is a bulk substrate, and given that it is a sustainable resource readily available in the worldwide market, its cost is significantly low. In this comparative study, it can also be observed that the current composite possesses a higher PC, removal efficiency (%), as well as equilibrium adsorption capacity (Q_e_) compared to the virgin GL as well as all other adsorbents. Therefore, it can be concluded that the resulting adsorbent is a comparable adsorbent material suitable for the removal of MB dye; however, further analyses such as studies on competitive and leaching effects are required in the near future.

## 5. Adsorption Performance of MnFe_2_O_4_/GL for Other Dyes Molecules

**Figure 10:**
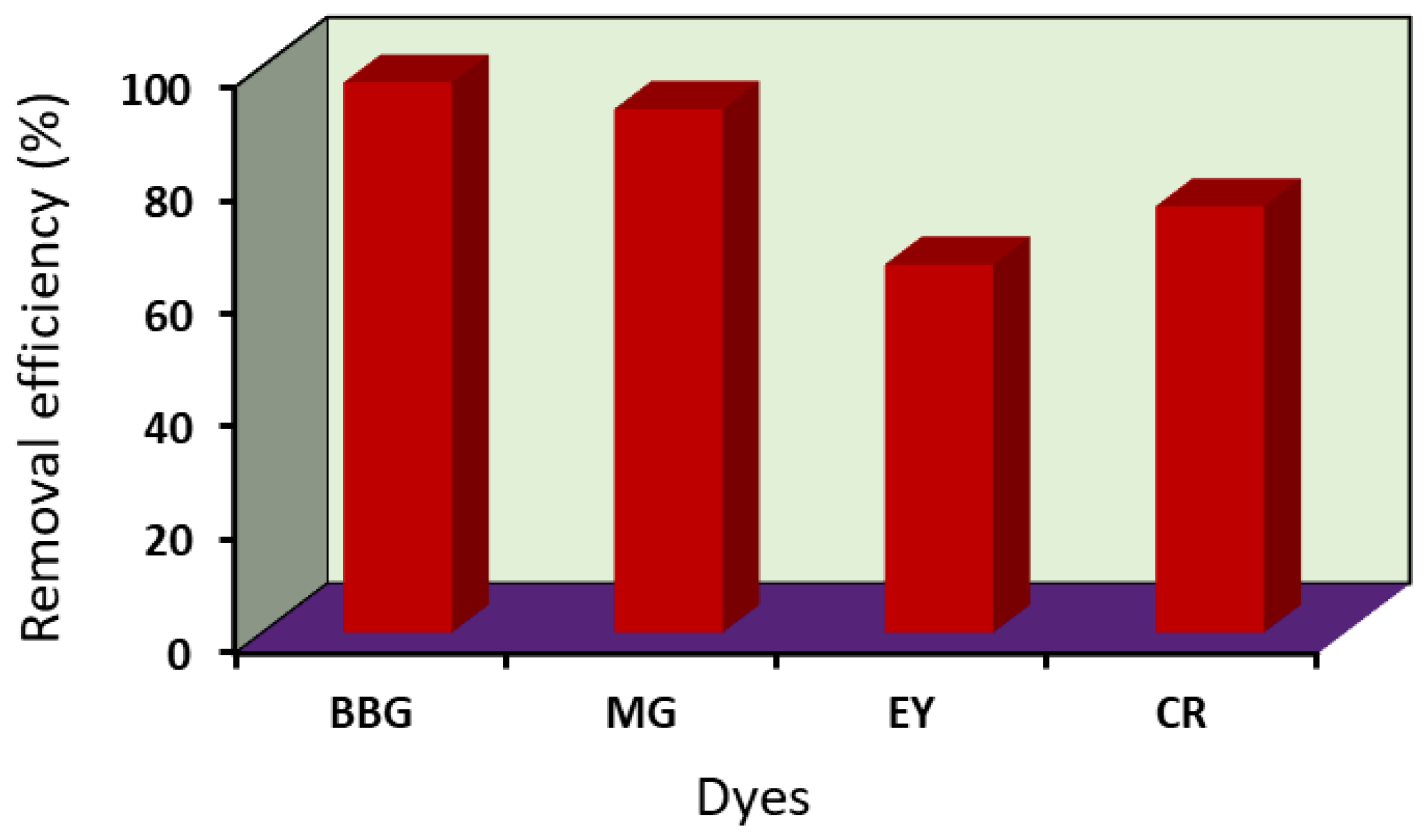
Adsorption performance of MnFe_2_O_4_/GL for other dyes.

In this study, the developed adsorbent, MnFe_2_O_4_/GL was also utilized for various other dyes such as Congo red (CR), Bismark brown G (BBG), Malachite green (MG), and Eosin Yellow (EY). The current investigation revealed that MnFe_2_O_4_/GL possesses significant potential for the removal of these pollutants as well. These results are depicted in Figure 10. Figure 10 clearly indicates that MnFe_2_O_4_/GL proved to be particularly effective for cationic dyes (BBG and MG). Under identical conditions (as used for the MB), the adsorbent achieved removal rates of ~97% for BBG and ~92% for MG; additionally, anionic dyes were also removed with varying degrees of efficiency (~70% for CR, 62% EY). These results underscore the impressive removal efficiency of the developed material, MnFe_2_O_4_/GL. However, more detailed studies are required in the future to elucidate the precise adsorption mechanisms involved in the adsorption of these specific dyes.

## 6. Conclusions

The conclusion of this study is that the current research has successfully synthesized a functionalized nanocomposite, formed through the electrostatic and non-electrostatic interactions between manganese ferrite and guava leaves. This nanocomposite was utilized to remove Methylene blue dye from water; specifically, a dosage of 2 g L^−1^ was capable of removing approximately ~99% of the dye within 90 min at an initial concentration of 10 mg L^−1^. This material demonstrated effective adsorption at a neutral pH, indicating its potential utility in water treatment applications. The adsorption of Methylene blue was found to be an exothermic process, followed by multilayer, heterogeneous adsorption, as evidenced by fitting the adsorption data to the Freundlich isotherm model. The adsorption kinetics followed a pseudo-second-order model, wherein intra-particle diffusion was identified as the rate-determining step. This nanocomposite proved to be reusable for up to five cycles, with only a 24% reduction in efficiency observed after the fifth cycle; this suggests that the composite material possesses significant potential for reusability. The efficiency of various dyes’ removal by the developed MnFe_2_O_4_/GL was also reported. In future studies, this material could be investigated for the removal of other metal ions, such as chromium and arsenic. Furthermore, its efficacy in natural water matrices could be evaluated. Emphasis should also be placed on investigating leaching effects and developing methods to remove the residual dye concentrations remaining in the solution post-adsorption; to this end, future research should also assess the photocatalytic degradation activity of this adsorbent material.

## Figures and Tables

**Table 1:** Absorption peaks at various wave numbers.

Order	Functional Groups	Wavenumber in GL	Wavenumber (cm^−1^) in MnFe_2_O_4_/GL Before MB Adsorption	Wavenumber (cm^−1^) in MnFe_2_O_4_/GL After MB Adsorption	Remarks
**1.**	-OH stretching	3326 cm^−1^	3291 cm^−1^	3280 cm^−1^	Absorbed water/hydroxyl groups in cellulosic surface of GL [43]
**2.**	-C-H stretching	2923 cm^−1^ (For -CH_3_) and 2847 cm^−1^ (For -CH_2_)	2923 cm^−1^ (For -CH_3_) and 2847 cm^−1^ (For -CH_2_)	2923 cm^−1^ (For -CH_3_) and 2847 cm^−1^ (For -CH_2_)	Presence of alkyl group suggesting carbon skeleton [43]
**3.**	N-H bend/Amide I	1613 cm^−1^	1572 cm^−1^	1577 cm^−1^	Presence of amide group in GL [52,55]
**4.**	C−O stretching	1395 cm^−1^	1362 cm^−1^	-	Suggests cellulose and other carbohydrates [55]
**5.**	H-C-O stretching, C-O-C stretching, and/or C-O stretching	1174 cm^−1^ and 1047 cm^−1^	1156 cm^−1^ and 1027 cm^−1^	1150 cm^−1^ and 1018 cm^−1^	
**6.**	C-O, C-C stretching/bending, and out of plane -C-H bending	950–800 cm^−1^	Absent	-	Suggests saccharides, glycosides, and lipids structure of leaves [55]
**7.**	M-O bond	Absent	471 cm^−1^	471 cm^−1^	Mn-O/Fe-O [51,56]

**Table 2:** Thermodynamics parameters.

Temperature (K)	∆G° (kJ mol^−1^)	∆H° (kJ mol^−1^)	∆S° (kJ mol^−1^ K^−1^)
303	−3.45	−47.34	−0.145
313	−1.75		
323	−0.55		

**Table 3:** The isotherm parameters for MB adsorption by MnFe_2_O_4_/GL.

Temp. (K)	Langmuir Model					Freundlich Model			
	*Q*_o_ (mg g^−1^)	b (L mg^−1^)	R_L_	R^2^	ARE	N	K_F_ (mg g^−1^)(L mg^−1^)^1/n^	R^2^	ARE
300	27.25	0.60	0.150	0.95	47.10	2.42	15.13	0.99	3.72
308	28.08	1.03	0.088	0.91	10.05	2.18	13.50	0.98	6.86
318	24.70	2.11	0.045	0.91	50.99	1.97	9.55	0.99	4.72

**Table 4:** Kinetics parameter for adsorption of MB by MnFe_2_O_4_/GL.

PFO Expt. Qe = 4.97 mg g^−1^			PSO Expt. Qe = 4.97 mg g^−1^			Elovich		
K_1_ (min^−1^)	Qe (mg g^−1^)	R^2^	k_2_ (g mg^−1^ min^−1^)	Qe (mg g^−1^)	R^2^	α (mg g^−1^ min^−1^)	β (g mg^−1^)	R^2^
0.006	8.50	0.98	0.123	5.36	0.99	6.07	0.66	0.98

**Table 5:** Results of WM plot.

Order	WM Model					
	First (Blue) Line			Second (Red) Line		
	Kd_1_ (mg g^−1^ min ^0.5^)	C_1_	R^2^	Kd_2_ (mg g^−1^ min ^0.5^)	C_2_	R^2^
1.	0.050	0.175	0.99	0.002	4.95	0.76

**Table 6:** (**a**) Performance evaluation of adsorbents used to remove MB from water at different concentrations. (**b**) Comparative performance evaluation of adsorbents used to remove MB from water. Adapted with permission from Siddiqui et al. [59], copyright (2025) MDPI.

(**a**)							
**Initial Concentrations** **(mg L^−1^)**			**Equilibrium Adsorption Capacity (Q_e_)**			**Partition Coefficient (L g^−1^)**	
**10**			4.97			91.9	
**15**			7.35			24.9	
**20**			9.78			22.1	
**25**			12.2			19.8	
**30**			14.5			16.7	
**35**			16.8			12.9	
**40**			19.2			12.1	
**45**			21.5			11.0	
(**b**)							
**Order**	**Adsorbent**	**Reaction Time** **(min)**	**Adsorption Dose (m)** **(g L^−1^)**	**Equilibrium Adsorption Capacity (mg g^−1^)**	**Removal Efficiency (%)**	**PC** **(L g^−1^)**	**Reference**
**1.**	MnFe_2_O_4_/BC	45	3	3.3	99.4	52.5	[51]
**2.**	Fe_2_O_3_-ZrO_2_/BC	60	3	3.3	99.4	55.2	[52]
**3.**	Ag/Ag_2_O/Zr O_2_/GL	60	2	4.9	99.0	50.6	[58]
**4.**	rGO-BC@ZrO_2_	90	2	4.9	99.0	51.4	[60]
**5.**	Fe_2_O_3_-SnO_2_/BC	90	2	4.9	97.9	23.9	[61]
**6.**	Fe_3_O_4_/BC	60	2	4.9	99.1	57.2	[59]
**7.**	GL	90	2	4.8	97.1	16.9	Current study
**8.**	MnFe_2_O_4_/GL	90	2	5.0	99.4	91.9	

## Data Availability

The data that support the findings of this study are available from the corresponding author, upon reasonable request.
